# Rapid and Quantitative Detection of Lung Cancer Biomarker ENOX2 Using a Novel Aptamer in an Electrochemical DNA-Based (E-DNA) Biosensor

**DOI:** 10.3390/bios13070675

**Published:** 2023-06-25

**Authors:** Mary Quansah, Lisa Fetter, Autumn Fineran, Haley V. Colling, Keaton Silver, Teisha J. Rowland, Andrew J. Bonham

**Affiliations:** 1 Department of Chemistry & Biochemistry, Metropolitan State University of Denver, Denver, CO 80204, USA; 2 Program in Biomolecular Science and Engineering, University of California Santa Barbara, Santa Barbara, CA 93106, USA; 3 Department of Molecular, Cellular, and Developmental Biology, University of Colorado, Boulder, CO 80309, USA

**Keywords:** aptamer, electrochemistry, cancer, ENOX2, biomarker, biosensor, E-DNA biosensor, cancer detection

## Abstract

To overcome early cancer detection challenges, diagnostic tools enabling more sensitive, rapid, and noninvasive detection are necessary. An attractive cancer target for diagnostic blood tests is human Ecto-NOX disulfide–thiol exchanger 2 (ENOX2), expressed in most human cancer types and regularly shed into blood sera. Here, we developed an electrochemical DNA-based (E-DNA) biosensor that rapidly detects physiologically relevant levels of ENOX2. To identify ENOX2-binding aptamers that could potentially be used in a biosensor, recombinantly expressed ENOX2 was used as a binding target in an oligonucleotide library pull-down that generated a highly enriched ENOX2-binding aptamer. This candidate aptamer sensitively bound ENOX2 via gel mobility shift assays. To enable this aptamer to function in an ENOX2 E-DNA biosensor, the aptamer sequence was modified to adopt two conformations, one capable of ENOX2 binding, and one with disrupted ENOX2 binding. Upon ENOX2 introduction, a conformational shift to the ENOX2 binding state resulted in changed dynamics of a redox reporter molecule, which generated a rapid, significant, and target-specific electrical current readout change. ENOX2 biosensor sensitivity was at or below the diagnostic range. The ENOX2 E-DNA biosensor design presented here may enable the development of more sensitive, rapid, diagnostic tools for early cancer detection.

## 1. Introduction

Cancer is the second leading cause of death in the United States, with lung cancer specifically being the primary cause of cancer deaths [1]. As ~50% of cancer cases are diagnosed at an advanced stage [2], when prognoses are poorer and there are more limited treatment options, early detection is essential for improving patient treatment options and survival rates [3]. A promising early detection biomarker is Ecto-NOX disulfide–thiol exchanger 2 (ENOX2, also referred to as tNOX) [4], which is detectable in the blood prior to observable clinical cancer symptoms [5]. Expression of the cell surface protein ENOX2 positively correlates with cell growth [5] and abnormal, neoplastic cell phenotypes, primarily in the lungs. Because these pre-invasive, early cancer cell types shed high levels of ENOX2 into blood, ENOX2 is an appealing biomarker to use for early, pre-symptomatic cancer detection—for lung and other cancer types—with blood-based diagnostic tools [5,6,7,8].

Using aptamers in an electrochemical DNA-based (E-DNA) biosensor is an appealing strategy for the development of a rapid, sensitive diagnostic tool for ENOX2 detection in patient blood samples. ENOX2 has been previously analyzed in diagnostic assays such as Western blot [9], RT-PCR-based, or PCR combined with chemiluminescence methods [10,11,12]. Such approaches to date have generally been time-consuming and required off-site lab support and analysis. Overall, these approaches have been limited, inefficient, and impractical for widespread, regular clinical adoption [7,8,12]. Compared to these approaches, E-DNA biosensors offer a much more rapid, on-site diagnostic solution, and the comparison of diagnostic parameters of E-DNA biosensors and other methods has been previously investigated [13,14]. E-DNA biosensors can provide real-time detection of biomarker targets, with previous successes including chemotherapeutic agents [15,16] and botulinum neurotoxin A [17], at biologically or clinically relevant concentrations. E-DNA biosensors can effectively function in solutions as adulterated and biologically complex as whole blood, soil [18], or live mammalian systems [15,16], making their adoption as diagnostic and analytical tools in a range of biological systems quite feasible.

To create an effective E-DNA biosensor, a target-specific aptamer is incorporated into an electrochemical platform that results in a measurable change in current upon biomarker target binding. Initially, oligonucleotide aptamers are screened—or identified from the literature—and tested for their ability to bind with high affinity and specificity to the target of interest. Next, a successful aptamer is incorporated into an E-DNA biosensor platform. In this platform, to detect the presence of the target, the primary readout employed is a change in electrochemical current. To achieve this readout, the E-DNA biosensor platform uses a gold disk electrode to which the selected aptamer is bound. Importantly, the selected aptamer is modified to enable it to exist in one of two conformations: (1) a conformation capable of target binding (the “binding state”) and (2) a conformation that is iso-energetically favorable but with disrupted target binding (the “non-binding state”). The aptamer is additionally modified to contain a redox reporter molecule that possesses a high electron transfer rate; here, we utilize methylthioninium chloride (i.e., methylene blue), a standard redox tag commonly used in E-DNA biosensors [15,18,19]. Upon introduction of the target to the completed E-DNA biosensor platform, changes in molecular dynamics (i.e., size, flexibility, diffusion rate) shift the aptamers to favor the binding state conformation, a conformation in which the redox reporter is a different distance from the gold disk electrode, resulting in a measurable change in current readout. This current change can be used to generate E-DNA biosensors with a “signal-on” or “signal-off” regime (increase or decrease in current readout upon target exposure, respectively) [20,21]. Hence, by incorporating aptamers into such an E-DNA biosensor platform, a rapid, real-time, sensitive readout is possible at clinically relevant target concentrations. 

Here, we present an aptamer-based E-DNA biosensor platform for detecting ENOX2. We used a bead-based aptamer selection strategy [22], coupled with high-throughput sequencing, to identify short oligonucleotide aptamers that potentially bind ENOX2 protein. After generating a fluorescently labeled version of the ENOX2-binding aptamer, the candidate was validated for sensitive, specific binding of ENOX2 in electrophoretic mobility shift assays. The validated aptamer was then modified via additions, deletions, or changes to the base sequence to create an E-DNA biosensor. The biosensor design produced a rapid signal-off response in a dose-responsive manner to ENOX2 in a physiologically relevant solution. The ENOX2 E-DNA biosensor design presented here holds promise as a path to developing a rapid, sensitive, early diagnostic tool for early lung cancer and other relevant cancer types.

## 2. Materials and Methods

Unless otherwise mentioned, all reagents were purchased from Sigma-Aldrich (St. Louis, MO, USA) and used as formulated.

### 2.1. Bead-Based Aptamer Selection

Recombinant human ENOX2 protein (Creative Biomart, Shirley, NY, USA) was biotinylated using the EZ-Link Sulfo-NHS-Biotinylation Kit (ThermoFisher, Waltham, MA, USA). Biotinylated ENOX2 protein was then bound to magnetic streptavidin-coated beads, Dynabeads M-270 Streptavidin (ThermoFisher), all following manufacturer protocols. Bead-bound ENOX2 protein was used for aptamer selection in the X-Aptamer Selection Kit (AM Biotechnologies, Houston, TX, USA), following the manufacturer protocol [22] for positive selection (pull-down of library oligonucleotides that bind the bead-bound ENOX2), negative selection (pull-down of off-target oligonucleotides by non-ENOX2 functionalized beads), and in-solution selection (pull-down of oligonucleotides by non-bound biotinylated ENOX2), followed by bead capture and magnetic pull-down. Selected oligonucleotides were validated via PCR (Figure S1). Abundance and identity of unique sequences were determined via high-throughput sequencing; the most highly enriched sequence was 5′-TTT TTT AGA TCC CTG TTC ATC TGT CTC GAG TGT TCT TTA ATG ATC TTT TT-3′ [22] (AM Biotechnologies).

### 2.2. ENOX2 Binding of Fluorescein-Labeled Putative Aptamer

**Figure 1:**
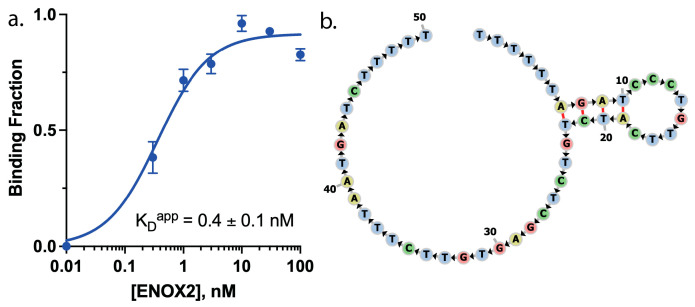
Verification of the ENOX2-binding aptamer. (**a**) Electrophoretic mobility shift assay (EMSA) analysis using an agarose gel (0.7%) with fluorescein-labeled putative ENOX2 binding aptamer shows sensitive ENOX2 binding, revealing an apparent K_D_ (K_D_^app^) of 0.4 ± 0.1 nM or 25.2 ± 6.3 ng/mL (gel image available in Figure S2). (**b**) Predicted secondary structure of the ENOX2-binding aptamer.

This enriched aptamer was synthesized with the addition of a 5′ fluorescein tag for experimental verification (IDT, Coralville, IA, USA). Electrophoretic mobility shift assays (EMSAs) were performed using agarose gels (0.7%) with tris-borate-ethylenediaminetetraacetic acid (TBE) (0.5×) consisting of Tris (65 mM), boric acid (22.5 mM), EDTA (1.25 mM), MgCl_2_ (0.5 mM), Tween-20 (0.005%), pH 8.3; fluorescein-labeled aptamer (1 nM); and recombinant ENOX2 protein (0 nM to 100 nM). Gels were imaged using a Gel Doc XR imaging system (Bio-Rad Laboratories, Inc., Hercules, CA, USA) and quantified using lane analysis with ImageJ 1.53k software (NIH, Bethesda, MD, USA) [23] (Figure 1a; image quantification data in Table S1). The secondary structure of the aptamer was predicted using RNAStructure [24] and visualized (Figure 1b) with Dash-Bio FornaContainer (Plotly Enterprises, Montreal, Canada).

### 2.3. Aptamer Design and Preparation

**Figure 2:**
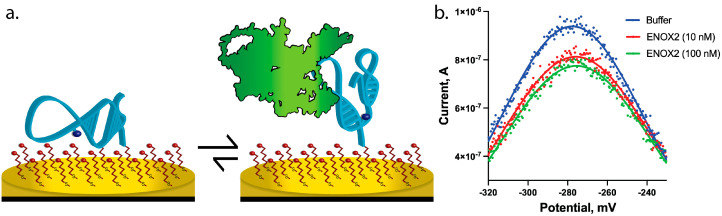
ENOX2 biosensor design. (**a**) Schematic of E-DNA biosensor design, showing both ENOX2 non-binding (**left**) and binding (**right**) conformations, with ENOX2 shown in green, biosensor in light blue, and methylene blue shown as a dark blue sphere; passivating layer in red is shown on gold electrode. Upon ENOX2 introduction and binding, changes in molecular dynamics shift the aptamer to favor the binding conformation. This conformational rearrangement is predicted to change the distance of the methylene blue from the gold disk electrode, resulting in a detectable signal change. (**b**) Square wave voltammetric (SWV) trace of the biosensor when incubated in PBS buffer alone to obtain a baseline measurement (blue), or PBS containing recombinant human ENOX2 protein (red (10 nM) or green (100 nM)), showing a methylene blue peak current at −275 mV that displays an ENOX2-responsive current decrease (data fit to Gaussian with GraphPad Prism).

The high-affinity ENOX2-binding aptamer (validated as described in Section 2.2) was converted into a functional biosensor aptamer by enabling a conformation-switching approach, which has been used successfully by our lab and others [25,26]. Specifically, this approach creates a measurable change in an electrochemical readout by using a modified aptamer that exists in two conformations, one in a target binding state and the other in an iso-energetically favorable but target non-binding state. Using this approach, we used computational modeling approaches to modify the oligonucleotide sequence of the aptamer, appending or removing nucleotides at the 5′ or 3′ ends, or changing internal bases not predicted to be involved in binding interactions [25]. The resultant aptamer displayed a two-state system, as described above, wherein one conformation adapted a structure capable of ENOX2 binding (the “binding state”), and the other, iso-energetically favorable conformation (determined via Quikfold Secondary Structure Prediction Routines [27]) displayed disrupted ENOX2 binding (the “non-binding state”) (Figure 2a), maximizing the detectable signal change [28]. This two-state sequence was further modified to enable binding to the gold disk electrode surface via the addition of a disulfide linker molecule to generate a 5′ gold-thiol bond with the electrode surface. Within this modified sequence, to incorporate a redox (i.e., electrochemically active) reporter molecule, a methylene blue molecule was appended to a thymine base. The final biosensor DNA sequence was 5′-(disulfide)-TAA AGA TCC CTG TTC ATC TTT (methylene blue) TCT CGA GTG TTC TTT AGA ACA ATG AAT CTT T-3′. This biosensor DNA sequence was synthesized via phosphoramidite column synthesis and purified via reverse-phase high-performance liquid chromatography (HPLC) by the manufacturer (Biosearch Technologies, Petaluma, CA, USA). The synthesized biosensor aptamer DNA was resuspended in ultrapure water to a final concentration of 100 µM, divided into 5 µL aliquots in microcentrifuge tubes, and stored at −20 °C until use (Section 2.4). 

### 2.4. Biosensor Design and Preparation

Gold disk electrodes (CH Instruments, Austin, TX, USA; 2 mm diameter) were electrochemically cleaned as previously described [29], with minor modifications. Specifically, electrodes were cleaned to remove organic surface contamination using repeated cyclic voltammetric scans in acid and base solutions to oxidize away surface impurities [29]. Such cleaning procedures are similarly used for the gold disk electrodes utilized in this study as well as for screen-printed thin film gold electrodes [30]. Unless otherwise specified, all steps were conducted at 25 °C. Briefly, the electrodes were first placed in sodium hydroxide (NaOH) (0.5 M) and cycled from −0.4 V to −1.35 V. Next, to oxidize and reduce contaminants on the gold surface, the electrodes were placed in sulfuric acid (H_2_SO_4_) (0.5 M) and held at 2.0 V for 5 s, and then held at 0.35 V for 10 s. Next, the electrodes were cycled from 0.35 V to 1.5 V for 25 scans at a linear scan rate of 4 V/s. To etch away any remaining contaminants, the electrodes were then placed in a solution of potassium chloride (KCl) (0.01 M) and H_2_SO_4_ (0.1 M) and cycled from 0.2 V to 1.5 V for 40 scans. A final cyclic voltammetry scan that cycled from −0.4 V to 0.1 V was performed in H_2_SO_4_ (0.05 M). The prepared electrode was lastly briefly washed with H_2_O (18 MΩ) prior to bonding with the aptamer.

To bind the prepared gold disk electrode to the biosensor DNA, the biosensor aptamer DNA (100 µM, synthesized as described in Section 2.3) was initially mixed 1:1 (5 µL of each component) with tris(2-carboxyethyl)phosphine hydrochloride (TCEP) (1 M) in a microcentrifuge tube and allowed to react in darkness to completion (≈30 min), acting to reduce disulfides to reactive thiols. The aptamer:TCEP mixture was then suspended in phosphate-buffered saline (PBS) pH 7.4, consisting of NaCl (137 mM), Na_2_HPO_4_ (10 mM), KH_2_PO_4_ (1.8 mM), KCl (2.7 mM), MgCl_2_ (1 mM), to a final volume of 100 µL, resulting in a 5 µM final concentration of activated DNA. To bind the biosensor DNA to the cleaned gold disk electrode, the electrode was submerged in this activated biosensor DNA solution and incubated for 45 min [29]. Previous studies optimized DNA aptamer concentration and incubation times, and the results from those studies guided the parameters used in this study [29,31]. When incubation time was shortened (<45 min), we observed no stable, measurable voltammetric signal. Similarly, alternative DNA concentrations were tested, and it was found that DNA aptamer concentrations above 20 µM or below 1 µM resulted in insufficiently stable voltammetric signal. To passivate the electrode surface from non-specific binding interactions, the electrode was then removed from DNA solution and submerged and incubated in 6-mercaptohexanol (300 µL total volume; 1 mM)(≈16 h at 4 °C); this binds to any unreacted electrode disk surface (via thiol-linkage), producing a passive hydroxyalkyl surface. 

### 2.5. Electrochemical and Control Parameters

**Figure 3:**
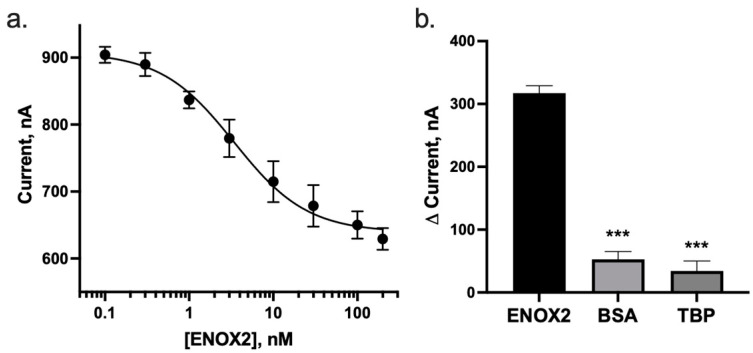
The ENOX2 E-DNA biosensor displayed robust performance in detecting ENOX2 in PBS. (**a**) Changes in peak current observed via SWV with titration of ENOX2 protein (ranging from 0.1 to 200 nM) displayed an apparent K_D_ (K_D_^app^) of 6 ± 3 nM or 378 ± 189 ng/mL. (**b**) Changes in current when the biosensor was exposed to ENOX2 were significantly greater than changes in current observed when the biosensor was exposed to off-target proteins, specifically BSA or TBP. Error bars indicate standard error of the mean. *** indicates *p*-value < 0.0001 for pairwise comparison to ENOX2 data.

Voltammetric data were collected using a WaveNano potentiostat (Pine Research Instrumentation, Durham, NC, USA) and their Aftermath software suite (version 1.6.10513) in square wave voltammetry (SWV) mode. Proteins tested with the prepared biosensor (prepared as described in Section 2.4) included: recombinant human ENOX2 protein (Creative Biomart, Shirley, NY, USA), bovine serum albumin (BSA), and recombinant TATA-binding protein (TBP), a transcription factor with known nonspecific affinity for DNA (previously purified by the lab [25]). When performing binding assays, the prepared biosensor was briefly rinsed with PBS, equilibrated in PBS (30 min), then allowed to equilibrate with each tested protein solution for 10 min, as this sensor displays rapid equilibration and stable response to target after ~5 min (Figure S3), which is in agreement with prior studies demonstrating this time to be sufficient for target equilibration [15,25,29]. Biosensors were scanned from −500 mV to 100 mV, with a 0.5 mV increment and 50 mV amplitude at 166 Hz. A frequency of 166 Hz was selected as it had been identified as producing the largest signal change upon addition of ENOX2 target (Table S2). Under these conditions, a peak in the current was observed at approximately −275 mV (Figure 2b), caused by the electron transfer from the methylene blue moiety of the biosensor [29]. Peak current was quantified via export of the data as a comma-separated-values file, which was then input into a custom, previously published peak-fitting program SWVAnyPeakFinder [15] (code available at https://github.com/Paradoxdruid/SWVAnyPeakFinder, accessed on 12 January 2022). This program identifies the peak in current and the horizontal baseline minimum current signal and performs baseline subtraction to yield a final adjusted peak current in a reproducible manner. Peak current data were analyzed using Prism 8 (GraphPad, version 8.4.0; La Jolla, CA, USA) with *n* > 3 and error bars depicting standard error of the mean (SEM) (Figure 3a). Apparent dissociation constants were obtained using Prism 8 with a nonlinear, three-parameter dose–response model based on the Langmuir isotherm adsorption model (Equation (1), where y is the adjusted peak current, a is the minimum response plateau, b is the top response plateau, K_D_ is the apparent dissociation constant, and x is the ENOX2 concentration).

y = a + (b − a)/(K_D_ + x) (1)

Limit of detection (LOD) was calculated using the methods of the Clinical and Laboratory Standards Institute [32] (Equation (2), where LOD is the limit of detection, σ is the standard deviation of the sample, and S is the slope of the calibration curve).

LOD = 3.3 σ/S(2)

## 3. Results

### 3.1. ENOX2-Binding Aptamer Screening

To generate a DNA aptamer sequence that targeted ENOX2, recombinant human ENOX2 protein was used as a selection target with the X-Aptamer Selection Kit, as described in Section 2.1 [22]. A highly enriched putative aptamer sequence, identified and sequenced through this selection process, was then synthesized with a 5′ fluorescein tag (Section 2.2) to interrogate ENOX2 binding function via EMSA analysis. This aptamer demonstrated effective ENOX2 binding via EMSA analysis (Figure 1a,b, and Table S1), with an apparent dissociation constant (K_D_^app^) of 0.4 ± 0.1 nM or 25.2 ± 6.3 ng/mL for ENOX2. Consequently, this aptamer was chosen for secondary conformation prediction analysis and further efforts.

### 3.2. ENOX2-Binding Aptamer Optimization to Enable Two Conformations

We used the ENOX2-binding aptamer identified in Section 3.1 as the starting sequence for our biosensor aptamer, modifying this sequence to exist in two conformations, an ENOX2-binding state, and an equally energetically favorable ENOX2-non-binding state, as described in Section 2.3. This strategy builds upon methods we have optimized for biosensors against a range of proteinaceous and small-molecule targets [17,25]. Briefly, each base in the ENOX2 aptamer sequence was categorized as essential or dispensable, based on the Quikfold predicted secondary structure including ENOX2 binding. Essential bases preserved for ENOX2 binding included bases in loops and the presence (but not identity) of bases required for proper spacing of predicted distances between loops (these disordered loops typically form the target binding regions of aptamers [25]). Bases dispensable for ENOX2 binding included those in stems and on the outer edges of the ENOX2 aptamer sequence. Dispensable bases were sequentially removed or replaced with new sequences, where bases outside of loops in the predicted structure needed to be at least partially complementary to the bases in the loops, generating new stems that can bind the loop bases, preventing them from forming target interactions and creating a non-binding conformation. Iterative designs were assessed using the Quikfold [33] secondary structure prediction tool to find a sequence that possessed both binding and non-binding folded states that were isoenergetic [17,25]. 

### 3.3. ENOX2 Binding Results in Detectable and Specific Peak Current Decrease

After optimizing the ENOX2-binding aptamer to exist in either a binding or non-binding state (Section 3.2), we made additional modifications to the aptamer sequence to function in an E-DNA biosensor system, as described in Section 2.3 and shown in Figure 2a. The aptamer sequence was modified to include a 5′ thiol for gold disk electrode binding, and an electrochemically active redox reporter molecule (methylene blue) to produce a detectable current signal when interrogated using square wave voltammetry (SWV). The reporter molecule possesses a high electron transfer rate and exchanges electrons with the gold disk electrode’s surface, resulting in a measurable current, even in buffer (PBS) alone. Under the conditions used for our biosensor testing, a peak current for the methylene blue was observed at −275 mV (Figure 2b and Table S3). Therefore, at this voltage (−275 mV), a conformational change between the binding and non-binding states of a successful E-DNA biosensor system would be expected to cause an increase or decrease in current. Using our E-DNA biosensor system, we indeed observed a detectable decrease in the peak current (typical of a “signal-off” response) when the system was titrated with samples containing either 10 nM or 100 nM ENOX2 protein in comparison to the sample with PBS buffer alone (Figure 2b).

To determine the limit of detection and effective range of our ENOX2 E-DNA biosensor, we tested it with increasing concentrations of ENOX2. We found that this biosensor detected ENOX2 with a limit of detection of 1 nM or 63 ng/mL, with an ~80-fold dynamic range of detection. This is common for this class of biosensors, which display a non-linear dose response [26]. The biosensors rapidly and reproducibly bound ENOX2 in physiologically relevant PBS (Figure 3a and Table S4). A 32% signal decrease was observed between the lowest (0.1 nM) to highest (200 nM) concentrations tested, with an apparent dissociation constant (K_D_^app^) of 6 ± 3 nM or 378 ± 189 ng/mL (R^2^ = 0.914). This apparent affinity is typical of a number of other E-DNA target sensitivities [17,18,25], and is sufficiently sensitive to measure clinically relevant ENOX2 concentrations, which are reported to be approximately 1.3–664 nM or 82–4.18 × 10^4^ ng/mL [9,34]. No significant current change was observed when biosensors were challenged with PBS alone, suggesting that the current decrease is specific to ENOX2 and a resultant aptamer conformation shifts upon binding ENOX2. 

The ENOX2 E-DNA biosensor was additionally tested for its ability to specifically bind ENOX2 by testing the binding of off-target proteins. For off-target proteins, we tested bovine serum albumin (BSA) and the TATA-binding protein (TBP) transcription factor. TBP was chosen to allow us to test a non-specific DNA association biosensor response. Upon challenging the biosensor with these off-target proteins, resultant changes in current produced by either the BSA (500 nM) or TBP (200 nM) was significantly less than the change in current produced by ENOX2 (*p*-value < 0.0001 for both pairwise two-tailed Student’s *t*-test comparisons) (Figure 3b and Table S5). This indicates that the observed peak current decrease is likely specific to ENOX2 binding and not due to non-specific protein interactions.

## 4. Discussion

To detect ENOX2 protein, here we developed an E-DNA biosensor that changed conformation upon ENOX2 binding, resulting in a detectable current signal decrease (a “signal-off” response) enabling sensitive and specific detection of ENOX2, a diagnostic biomarker of lung cancer and other cancer types. The biosensor was found to reproducibly elicit a typical signal-off response, which is common for this class of biosensors [20,21]. It was also found to be sufficiently sensitive to detect ENOX2 in a physiologically relevant buffer (PBS) well below the clinically relevant range. Specifically, the K_D_^app^ observed here of 6 ± 3 nM (or 378 ± 189 ng/mL) is within the range of levels reported in the sera of presenting patients, reported as approximately 1.3–664 nM (or 82–4.18 × 10^4^ ng/mL) [9,34] (Figure 3a). A limitation of the current study and biosensor design is that a relatively large error was observed (Figure 3a). We believe this was due to instrumentation limitations (unfiltered noise and decreased resolution at the µA level) and baseline drift of the biosensor system. While the WaveNano potentiostat used in this study is representative of available low-cost, field portable instruments, with a single low-pass filter and resolution of 1.7 nA, the use of higher-end potentiostat instruments available offering improved resolution and filtering would likely result in reduced error [15,28]. Others have also improved signals by collecting dual measurements at both an active and inactive frequency, known as a kinetic differential measurement [15,16]. We believe that future work incorporating such drift reduction techniques and utilizing higher-grade instrumentation with increased resolution would significantly reduce the observed error. 

Our biosensor additionally showed a rapid response and high specificity. The response rate was under 10 min for complete measurement. High specificity was also demonstrated, as when the biosensor was challenged with off-target proteins (BSA and TBP), any change in peak current was significantly less than observed peak current changes for testing the biosensor with ENOX2 (Figure 3b). 

Overall, the E-DNA biosensor approach presented here presents significant advantages that may advance the diagnostic capabilities for early cancer detection through ENOX2. These advantages include the ability to function with inexpensive and handheld potentiometric detectors [35], which enables the potential for adoption in clinical settings. However, the most significant challenge is a roadmap to clinical validation of such biosensors; recently, several groups have demonstrated more robust E-DNA biosensors in relevant systems, such as ambulatory animals [15,16], but work remains in conducting patient trials and commercialization. A future direction for these efforts is validating this and similar derivative E-DNA sensors in complex media. Such efforts are likely to be successful as E-DNA sensors in general have shown reproducible function in blood, soil, and even alcoholic beverages [15,18]. Despite those previously reported successes in complex media, clinical verification of the utility of this and similar E-DNA sensors is a critical future direction. As those challenges become addressed, it will facilitate the rapid adoption of a wide range of aptamer-based systems, such as the ENOX2 biosensor presented here, into multiple medical diagnostic applications.

## 5. Conclusions

ENOX2 is a cancer biomarker of high clinical importance for its potential in both diagnostics and therapeutic targeting, but efficient and sensitive clinical tools for ENOX2 detection are currently limited, requiring off-site testing and dedicated lab environments. The approach demonstrated here, generating and screening highly sensitive DNA aptamers to identify ENOX2-specific binders and then incorporating a select aptamer into an E-DNA biosensor system, supports the growing use of E-DNA biosensors as broadly applicable diagnostic tools for a range of biomarkers and other molecular targets. Furthermore, the ENOX2 biosensor presented here displays sensitivity more than sufficient for clinical diagnostics, as well as an ENOX2-specific response in physiologically relevant buffer conditions. In future efforts, we aim to apply recently proven approaches to characterize this biosensor’s function in complex, physiologically relevant media conditions, such as unprocessed whole bovine or human blood, to further validate the utility of this biosensor for clinical diagnostic applications. These efforts would contribute towards establishing E-DNA biosensors as a viable strategy for medical and environmental diagnostics, particularly for early cancer screening efforts targeting early ENOX2 detection.

## Data Availability

The data presented in this study are available in the manuscript and the Supplementary Materials.

## Supplementary Materials

The following supporting information can be downloaded at: https://www.mdpi.com/article/10.3390/bios13070675/s1, Figure S1: PCR pool verification of aptamer; Figure S2: Gel image of EMSA; Figure S3: signal response over time; Table S1: Image quantification data of electrophoretic mobility shift assay for Figure 1a. Table S2: SWV frequency optimization; data presented in this study. Table S3: voltametric data for Figure 2b. Table S4: Normalized voltametric peak height titration data for Figure 3a. Table S5: voltametric data for off-target analysis for Figure 3b.
