# Supplementary material for: Rapid and Quantitative Detection of Lung Cancer Biomarker ENOX2 Using a Novel Aptamer in an Electrochemical DNA-Based (E-DNA) Biosensor
Source: Biosensors (Basel). 2023 Jun 25;13(7):675. doi: 10.3390/bios13070675 (PMC10377175; doi:10.3390/bios13070675)
Supplement: Supplementary file 1 [file biosensors-13-00675-s001.zip › biosensors-2421836-supplementary.pdf]

Supplementary Data for "Rapid and quantitative detection of lung cancer biomarker ENOX2 using a novel aptamer in an electrochemical DNA-based (E-DNA) biosensor".

Mary Quansah, Lisa Fetter, Autumn Fineran, Haley V. Colling, Keaton Silver, Teisha J. Rowland, and Andrew J. Bonham

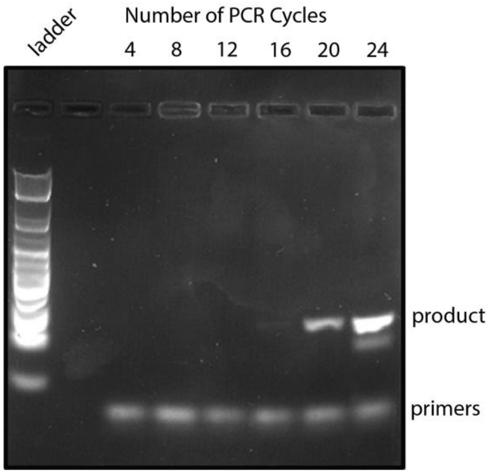

Figure S1. PCR Pool validation of ENOX2 binding aptamer. Assay performed with 3% agarose gel with 0.5X TBE; 50bp ladder (New England Biolabs, Ipswich, MA), candidate aptamer pools, and common end primers from the X-Aptamer Selection Kit (AM Biotechnologies, Houston, TX, USA). PCR amplification cycle number was varied to identify minimum cycle number for efficient amplification.

Table S1. Image quantification data of electrophoretic mobility shift assay **Figure 1a**.

[ENOX2], nM	Replicate #1	Replicate #2	Replicate #3
0.3	0.31606999	0.31276167	0.51812476
1	0.66863994	0.66730013	0.810444
3	0.74604973	0.74344432	0.87063023
10	0.99202746	0.89343431	1
30	0.90899324	0.91183724	0.96341463
100	0.79681615	0.80477255	0.87804878

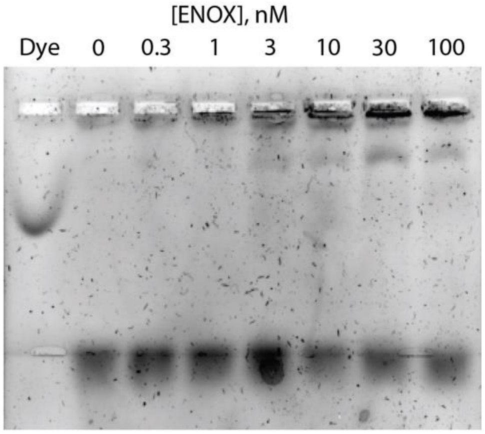

Figure S2. Electrophoretic mobility shift assay of ENOX2 binding aptamer. Assay performed using 0.7% agarose gel with 0.5X TBE; fluorescein-labeled aptamer (1 nM); recombinant ENOX2 protein (0 nM to 100 nM).

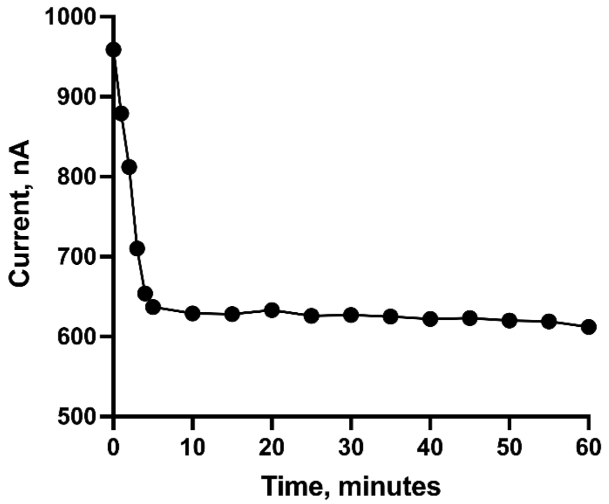

Figure S3. The biosensor rapidly (within 5 min) equilibrates to a final signal plateau when incubated with the ENO2 target protein. Changes in peak current over time were measured via SWV following addition of 200 nM ENOX2 protein (at 0 min). A stable response (>90% signal recorded) was demonstrated after 5 min. Based on these data, 10 min was used as the incubation time with ENOX2 protein for our biosensor.

Frequency, Hz	Blank Response, A	ENOX2 Response, A	Signal Change, %
10	1.88E-07	2.15E-07	14%
33	5.15E-07	5.69E-07	11%
63	7.44E-07	6.65E-07	-11%
100	7.25E-07	5.17E-07	-29%
166	9.30E-07	3.49E-07	**-62%**
250	1.52E-06	1.19E-06	-22%
1000	2.30E-06	2.00E-06	-13%

Table S2. Signal gain optimization by different square-wave voltammetry (SWV) frequency. The largest signal change upon ENOX2 target addition was observed with a SWV frequency of 166 Hz, which was consequently selected as the interrogation frequency for the biosensor.

Below is the data used to generate the figures in the article text.

Table S3. voltametric data for **Figure 2b**.

Potential (V)	Buffer	ENOX (10 nM)	ENOX (100 nM)
-0.40017	2.81e-007	3.06e-007	2.91e-007
-0.39945	3.02e-007	3.02e-007	3.35e-007
-0.39893	2.95e-007	3.34e-007	3.08e-007
-0.39854	2.75e-007	3.05e-007	3.3e-007
-0.39786	2.67e-007	3.13e-007	3.25e-007
-0.39744	3e-007	3.28e-007	3.07e-007
-0.3969	2.72e-007	3.18e-007	2.74e-007
-0.39645	2.64e-007	3.32e-007	3.47e-007
-0.3958	2.45e-007	3.28e-007	3.22e-007

-0.39541	2.41e-007	2.95e-007	3.12e-007
-0.39498	2.72e-007	3.07e-007	3.41e-007
-0.39442	2.61e-007	3.35e-007	3.01e-007
-0.39396	2.78e-007	3.12e-007	3.09e-007
-0.39341	2.64e-007	2.77e-007	3.1e-007
-0.39298	2.22e-007	3.05e-007	2.73e-007
-0.39254	2.53e-007	2.81e-007	3.03e-007
-0.39197	2.45e-007	2.99e-007	2.93e-007
-0.39152	2.78e-007	3.19e-007	2.78e-007
-0.39091	2.73e-007	3.27e-007	3.11e-007
-0.39045	3.06e-007	3.25e-007	2.78e-007
-0.38993	2.7e-007	3.25e-007	3.22e-007
-0.38956	2.72e-007	3.26e-007	3e-007
-0.38886	2.77e-007	2.96e-007	3.19e-007
-0.38837	2.47e-007	2.8e-007	2.86e-007
-0.38795	2.88e-007	2.95e-007	2.91e-007
-0.38727	2.45e-007	2.88e-007	2.88e-007
-0.38689	2.55e-007	2.81e-007	3.25e-007
-0.38644	2.55e-007	2.86e-007	3.1e-007
-0.38593	2.37e-007	3.05e-007	2.85e-007
-0.38549	2.86e-007	3.03e-007	2.95e-007
-0.38496	2.78e-007	2.52e-007	2.94e-007
-0.3844	2.45e-007	3.07e-007	2.82e-007
-0.38391	2.67e-007	2.69e-007	2.91e-007
-0.38344	2.3e-007	2.6e-007	2.8e-007
-0.38289	2.31e-007	3.05e-007	2.88e-007
-0.3824	2.51e-007	3.02e-007	2.73e-007
-0.38187	2.53e-007	2.74e-007	2.84e-007
-0.38163	2.55e-007	2.2e-007	3.01e-007
-0.38103	2.6e-007	3.12e-007	2.93e-007
-0.38043	2.63e-007	2.98e-007	3.02e-007
-0.37996	2.56e-007	2.96e-007	3e-007
-0.37935	2.49e-007	2.79e-007	3.17e-007
-0.37887	2.55e-007	2.64e-007	2.63e-007
-0.37832	2.29e-007	3.07e-007	2.3e-007
-0.37779	2.71e-007	2.62e-007	3.28e-007
-0.37763	2.61e-007	2.67e-007	3.07e-007
-0.37691	2.52e-007	3.14e-007	2.92e-007
-0.37657	2.56e-007	2.99e-007	3.05e-007
-0.37594	2.62e-007	2.64e-007	2.91e-007
-0.37539	2.43e-007	2.78e-007	2.94e-007
-0.375	2.62e-007	2.82e-007	2.7e-007
-0.37443	2.44e-007	3.01e-007	2.9e-007

-0.37394	2.41e-007	2.92e-007	2.35e-007
-0.37328	2.47e-007	3.03e-007	2.62e-007
-0.37299	2.45e-007	2.68e-007	2.69e-007
-0.37235	2.68e-007	2.5e-007	2.87e-007
-0.37192	2.73e-007	2.91e-007	2.78e-007
-0.37149	2.2e-007	3.12e-007	2.78e-007
-0.3708	2.54e-007	2.43e-007	3.16e-007
-0.37033	2.59e-007	2.83e-007	2.57e-007
-0.36999	2.44e-007	2.96e-007	2.44e-007
-0.36936	2.58e-007	2.65e-007	2.77e-007
-0.36887	2.65e-007	2.8e-007	3.02e-007
-0.36834	2.27e-007	2.89e-007	2.76e-007
-0.36775	2.61e-007	2.88e-007	2.62e-007
-0.36726	2.41e-007	2.92e-007	2.81e-007
-0.36675	2.08e-007	2.84e-007	2.83e-007
-0.36634	2.57e-007	2.69e-007	2.97e-007
-0.36589	2.65e-007	2.89e-007	2.47e-007
-0.36535	2.68e-007	2.46e-007	3.11e-007
-0.3648	2.32e-007	3.06e-007	2.37e-007
-0.36446	2.62e-007	2.7e-007	2.35e-007
-0.36394	2.44e-007	2.64e-007	3.04e-007
-0.36333	2.43e-007	2.72e-007	2.83e-007
-0.36293	2.71e-007	2.65e-007	2.84e-007
-0.36236	2.5e-007	2.57e-007	2.99e-007
-0.36191	2.5e-007	2.94e-007	2.57e-007
-0.36144	2.6e-007	3.02e-007	2.69e-007
-0.36084	2.58e-007	2.89e-007	2.83e-007
-0.3604	2.64e-007	2.88e-007	2.64e-007
-0.35981	2.41e-007	2.91e-007	2.85e-007
-0.35946	2.31e-007	2.82e-007	2.91e-007
-0.35878	2.45e-007	2.58e-007	2.75e-007
-0.35839	2.62e-007	2.73e-007	3.16e-007
-0.35789	2.78e-007	2.7e-007	2.92e-007
-0.35748	2.45e-007	2.79e-007	2.66e-007
-0.35694	2.46e-007	2.79e-007	3.3e-007
-0.35653	2.41e-007	2.73e-007	2.63e-007
-0.35592	2.67e-007	2.9e-007	2.83e-007
-0.35529	2.53e-007	2.89e-007	2.74e-007
-0.35479	2.67e-007	2.88e-007	2.95e-007
-0.35426	2.6e-007	2.97e-007	2.99e-007
-0.35389	2.67e-007	2.49e-007	2.89e-007
-0.35337	2.57e-007	2.92e-007	2.77e-007
-0.35277	2.89e-007	2.73e-007	3.1e-007

-0.35245	2.8e-007	2.7e-007	2.87e-007
-0.35176	2.74e-007	2.78e-007	2.9e-007
-0.3514	2.67e-007	2.87e-007	2.9e-007
-0.35089	2.72e-007	2.71e-007	2.98e-007
-0.35031	2.58e-007	2.91e-007	2.77e-007
-0.34989	2.87e-007	2.93e-007	2.86e-007
-0.34939	2.91e-007	2.86e-007	3.04e-007
-0.34879	2.91e-007	2.7e-007	2.53e-007
-0.34833	2.94e-007	3.17e-007	2.8e-007
-0.34791	2.96e-007	2.97e-007	2.95e-007
-0.34733	2.94e-007	2.97e-007	3.03e-007
-0.34691	2.89e-007	3.28e-007	2.9e-007
-0.34645	2.93e-007	2.85e-007	2.69e-007
-0.34584	3.1e-007	3.01e-007	2.69e-007
-0.34527	3.13e-007	2.94e-007	3.07e-007
-0.345	2.68e-007	2.71e-007	3.12e-007
-0.34441	2.97e-007	2.8e-007	2.86e-007
-0.34398	3.05e-007	2.89e-007	3e-007
-0.34337	3.02e-007	2.87e-007	3.11e-007
-0.34297	2.99e-007	3.05e-007	2.99e-007
-0.34236	2.99e-007	3.07e-007	3.16e-007
-0.34196	3.21e-007	2.95e-007	3.19e-007
-0.34136	2.67e-007	3.32e-007	3.07e-007
-0.34085	3.25e-007	3.43e-007	3.01e-007
-0.34039	3.23e-007	2.72e-007	3.36e-007
-0.33983	3.45e-007	2.97e-007	2.94e-007
-0.33939	3.43e-007	3.4e-007	3.13e-007
-0.33891	3.26e-007	3.12e-007	3.45e-007
-0.3384	3.02e-007	3.07e-007	2.93e-007
-0.3378	3.13e-007	3.37e-007	3.25e-007
-0.33734	3.46e-007	3.22e-007	2.79e-007
-0.33679	3.09e-007	3.27e-007	3.38e-007
-0.33631	3.23e-007	2.94e-007	2.99e-007
-0.33583	3.09e-007	3.52e-007	3.02e-007
-0.33531	3.3e-007	3.21e-007	2.94e-007
-0.33477	3.6e-007	3.59e-007	3.01e-007
-0.33439	3.59e-007	3.58e-007	3.68e-007
-0.33389	3.57e-007	2.86e-007	3.53e-007
-0.33335	3.57e-007	3.44e-007	3.46e-007
-0.33282	3.56e-007	3.4e-007	3.03e-007
-0.33245	4e-007	3.75e-007	3.31e-007
-0.33181	3.59e-007	3.51e-007	3.15e-007
-0.33132	3.91e-007	3.63e-007	3.37e-007

-0.33087	3.33e-007	3.7e-007	3.29e-007
-0.33048	3.74e-007	3.71e-007	3.6e-007
-0.32983	3.79e-007	3.66e-007	3.25e-007
-0.32942	4.16e-007	3.94e-007	3.74e-007
-0.32885	3.78e-007	4e-007	3.63e-007
-0.32842	3.99e-007	3.38e-007	3.71e-007
-0.32795	3.92e-007	3.95e-007	3.76e-007
-0.32738	3.9e-007	3.85e-007	3.29e-007
-0.32679	3.94e-007	4.12e-007	3.7e-007
-0.32632	4.22e-007	3.82e-007	3.86e-007
-0.32592	4.47e-007	3.82e-007	3.69e-007
-0.32535	4.34e-007	3.48e-007	3.78e-007
-0.32482	4.09e-007	4.31e-007	3.7e-007
-0.32431	4.52e-007	4.15e-007	3.7e-007
-0.32382	4e-007	4.05e-007	3.7e-007
-0.32331	4.27e-007	4.04e-007	3.91e-007
-0.32279	4.58e-007	4.1e-007	4.03e-007
-0.32247	4.44e-007	4.6e-007	4.09e-007
-0.32182	4.86e-007	4.22e-007	3.97e-007
-0.32132	4.77e-007	4.52e-007	4.02e-007
-0.32065	4.72e-007	4.3e-007	4.22e-007
-0.32029	4.6e-007	4.13e-007	4.1e-007
-0.31985	4.79e-007	4.05e-007	4.27e-007
-0.31934	4.73e-007	4.3e-007	3.81e-007
-0.31874	5.03e-007	4.42e-007	4.19e-007
-0.31826	5.33e-007	4.38e-007	4.21e-007
-0.31778	5.15e-007	4.59e-007	3.94e-007
-0.3173	5.32e-007	4.73e-007	4.21e-007
-0.31681	5.44e-007	4.95e-007	4.42e-007
-0.31633	5.7e-007	4.65e-007	4.25e-007
-0.31578	5.34e-007	4.54e-007	4.29e-007
-0.31519	5.45e-007	5.25e-007	4.36e-007
-0.31482	5.59e-007	4.85e-007	4.53e-007
-0.31438	5.22e-007	4.74e-007	4.65e-007
-0.31369	5.21e-007	4.5e-007	4.93e-007
-0.31325	5.4e-007	4.93e-007	4.66e-007
-0.31279	5.92e-007	4.81e-007	4.85e-007
-0.31239	5.66e-007	5.22e-007	4.76e-007
-0.31173	6.07e-007	5.38e-007	4.85e-007
-0.31131	5.9e-007	4.69e-007	4.83e-007
-0.31087	5.77e-007	4.97e-007	4.92e-007
-0.31025	6.36e-007	5.31e-007	5.09e-007
-0.30982	6.1e-007	5.43e-007	5.34e-007

-0.30921	6.07e-007	5.31e-007	5.14e-007
-0.3087	6.29e-007	5.49e-007	4.96e-007
-0.30834	6.27e-007	5.34e-007	5.51e-007
-0.3077	6.31e-007	5.54e-007	5.25e-007
-0.30716	6.39e-007	5.75e-007	5.18e-007
-0.30679	6.75e-007	5.62e-007	5.7e-007
-0.30631	6.68e-007	5.72e-007	5.31e-007
-0.30584	6.95e-007	5.97e-007	5.75e-007
-0.30523	6.76e-007	6.02e-007	5.51e-007
-0.30466	6.84e-007	6.16e-007	5.63e-007
-0.30427	7.04e-007	5.98e-007	5.47e-007
-0.30371	7.04e-007	5.86e-007	5.87e-007
-0.30319	7.23e-007	6.38e-007	5.65e-007
-0.30272	7.27e-007	6.36e-007	5.59e-007
-0.30226	7.3e-007	6.16e-007	6.08e-007
-0.30162	7.37e-007	6.29e-007	5.93e-007
-0.30144	7.28e-007	6.49e-007	5.97e-007
-0.30074	7.42e-007	6.59e-007	6.09e-007
-0.30026	7.45e-007	6.75e-007	6.13e-007
-0.29982	7.58e-007	6.6e-007	6.28e-007
-0.2993	7.66e-007	6.66e-007	5.91e-007
-0.29886	7.73e-007	6.79e-007	6.52e-007
-0.2983	7.98e-007	6.87e-007	6.41e-007
-0.29781	8.12e-007	6.95e-007	6.55e-007
-0.29732	7.84e-007	7.25e-007	6.75e-007
-0.29672	8.32e-007	6.87e-007	6.65e-007
-0.29628	8.16e-007	7.01e-007	6.48e-007
-0.29581	8.26e-007	7.08e-007	6.5e-007
-0.29529	8.26e-007	6.72e-007	6.67e-007
-0.29495	8.2e-007	7.06e-007	6.39e-007
-0.29434	8.27e-007	6.77e-007	7.18e-007
-0.29384	8.4e-007	7.74e-007	6.51e-007
-0.29335	8.51e-007	7.29e-007	6.79e-007
-0.29272	8.48e-007	7.27e-007	6.95e-007
-0.29229	8.57e-007	7.18e-007	6.96e-007
-0.29178	8.95e-007	6.89e-007	6.97e-007
-0.29126	8.64e-007	7.48e-007	6.96e-007
-0.29076	8.64e-007	7.51e-007	7.27e-007
-0.29033	8.97e-007	7.28e-007	7.12e-007
-0.28971	8.94e-007	7.58e-007	7.16e-007
-0.28928	9.11e-007	7.8e-007	7.19e-007
-0.28885	9.16e-007	7.46e-007	6.9e-007
-0.28831	9e-007	7.07e-007	6.91e-007

-0.28777	9.25e-007	7.91e-007	7.24e-007
-0.28721	9e-007	7.9e-007	7.4e-007
-0.28671	8.85e-007	7.83e-007	7.67e-007
-0.28619	9.19e-007	7.6e-007	7.39e-007
-0.28567	9.05e-007	7.89e-007	7.42e-007
-0.28526	9.37e-007	8.11e-007	7.67e-007
-0.28478	9.32e-007	7.93e-007	7.68e-007
-0.28424	9.27e-007	7.75e-007	7.36e-007
-0.28376	9.78e-007	7.87e-007	7.77e-007
-0.28326	9.17e-007	8.02e-007	7.71e-007
-0.28286	9.35e-007	8.17e-007	7.7e-007
-0.28226	9.03e-007	8.12e-007	7.98e-007
-0.28182	9.6e-007	7.99e-007	7.79e-007
-0.2812	9.59e-007	8.36e-007	7.55e-007
-0.28067	9.3e-007	7.93e-007	7.68e-007
-0.2803	9.68e-007	8.28e-007	7.96e-007
-0.2797	9.13e-007	8e-007	7.77e-007
-0.2792	9.33e-007	8.37e-007	7.71e-007
-0.27873	9.29e-007	8.28e-007	8.01e-007
-0.27833	9.6e-007	8.21e-007	7.94e-007
-0.27761	9.29e-007	8.01e-007	7.91e-007
-0.27722	9.4e-007	8.1e-007	7.9e-007
-0.27676	9.14e-007	8.48e-007	8.04e-007
-0.27626	9.57e-007	8.3e-007	7.55e-007
-0.2758	9.67e-007	8.55e-007	8e-007
-0.27533	9.45e-007	8.02e-007	7.71e-007
-0.27469	9.35e-007	7.89e-007	7.95e-007
-0.2743	9.72e-007	7.89e-007	7.92e-007
-0.27374	9.49e-007	8.24e-007	7.92e-007
-0.27317	9.79e-007	8.21e-007	8.01e-007
-0.27281	9.41e-007	8.21e-007	7.85e-007
-0.2722	9.05e-007	8.21e-007	7.83e-007
-0.27169	9.3e-007	8.28e-007	7.8e-007
-0.27115	9.19e-007	8.22e-007	8e-007
-0.27069	9.43e-007	8.41e-007	7.74e-007
-0.27029	9.03e-007	8.27e-007	7.56e-007
-0.2696	9.1e-007	8.25e-007	7.59e-007
-0.26907	9.21e-007	7.93e-007	8.07e-007
-0.26871	9.17e-007	8.27e-007	7.65e-007
-0.2683	9e-007	7.93e-007	7.34e-007
-0.26772	8.84e-007	8.01e-007	7.32e-007
-0.26728	9.14e-007	8.07e-007	7.44e-007
-0.26685	9.33e-007	7.96e-007	7.5e-007

-0.26632	9.09e-007	7.55e-007	7.64e-007
-0.26565	8.84e-007	8e-007	7.55e-007
-0.26529	9.2e-007	7.66e-007	7.23e-007
-0.26481	8.9e-007	8.03e-007	7.39e-007
-0.26433	8.75e-007	7.56e-007	7.48e-007
-0.26369	8.97e-007	8.06e-007	7.24e-007
-0.26329	8.48e-007	7.7e-007	7.35e-007
-0.26271	8.23e-007	7.47e-007	7.17e-007
-0.2622	8.63e-007	7.47e-007	7.16e-007
-0.26166	8.56e-007	7.51e-007	7.01e-007
-0.2613	8.62e-007	7.42e-007	7.33e-007
-0.26077	8.11e-007	7.58e-007	7.15e-007
-0.26022	8.2e-007	7.48e-007	7.26e-007
-0.25955	8.26e-007	7.45e-007	7.25e-007
-0.25918	8.3e-007	7.48e-007	6.89e-007
-0.25884	8.01e-007	7.16e-007	6.84e-007
-0.25828	7.71e-007	7.36e-007	6.67e-007
-0.25757	7.92e-007	7.05e-007	6.75e-007
-0.25717	8.06e-007	6.93e-007	6.77e-007
-0.25677	7.71e-007	7.09e-007	6.84e-007
-0.25607	7.81e-007	6.92e-007	6.82e-007
-0.2556	8.04e-007	7.09e-007	6.96e-007
-0.25524	7.46e-007	6.72e-007	6.4e-007
-0.25486	7.31e-007	6.75e-007	6.73e-007
-0.2543	7.2e-007	6.58e-007	6.64e-007
-0.25359	7.23e-007	6.47e-007	6.54e-007
-0.25313	7.41e-007	6.79e-007	6.49e-007
-0.25268	7.37e-007	6.46e-007	6.13e-007
-0.25229	7.03e-007	6.37e-007	6.19e-007
-0.25175	6.92e-007	6.59e-007	6.09e-007
-0.2512	6.59e-007	6.45e-007	6.45e-007
-0.25076	6.94e-007	6.51e-007	6.21e-007
-0.25016	6.69e-007	6.36e-007	6.38e-007
-0.24968	6.94e-007	6.42e-007	5.69e-007
-0.2491	6.52e-007	6.25e-007	6e-007
-0.24876	6.75e-007	6.14e-007	6.09e-007
-0.24828	6.33e-007	6.19e-007	5.64e-007
-0.24764	6.29e-007	6.29e-007	5.94e-007
-0.24725	6.42e-007	6.22e-007	5.78e-007
-0.24688	6.76e-007	5.69e-007	5.73e-007
-0.24624	6.31e-007	5.93e-007	5.57e-007
-0.24576	6.32e-007	5.8e-007	5.57e-007
-0.24507	5.9e-007	5.84e-007	5.48e-007

-0.24474	6.08e-007	5.66e-007	5.57e-007
-0.24427	6.15e-007	5.55e-007	5.52e-007
-0.24361	5.96e-007	5.61e-007	5.48e-007
-0.24334	5.71e-007	5.48e-007	5.38e-007
-0.24269	5.7e-007	5.35e-007	5.28e-007
-0.24214	5.87e-007	5.34e-007	5.32e-007
-0.24178	5.59e-007	5.41e-007	4.72e-007
-0.24125	5.59e-007	5.41e-007	5.28e-007
-0.24074	5.71e-007	5.44e-007	5.07e-007
-0.24032	5.64e-007	4.94e-007	4.62e-007
-0.23975	5.09e-007	5.06e-007	4.67e-007
-0.23922	5.21e-007	5.29e-007	4.67e-007
-0.23872	5.03e-007	4.9e-007	4.64e-007
-0.2383	5.68e-007	4.96e-007	4.62e-007
-0.23781	4.89e-007	4.92e-007	4.29e-007
-0.23731	5.07e-007	4.26e-007	4.61e-007
-0.23676	4.73e-007	4.35e-007	4.69e-007
-0.23625	4.87e-007	4.94e-007	4.59e-007
-0.23582	4.89e-007	4.9e-007	4.58e-007
-0.23526	4.48e-007	4.39e-007	4.54e-007
-0.23477	4.94e-007	4.64e-007	4.57e-007
-0.23423	4.97e-007	4.36e-007	4.6e-007
-0.23378	4.37e-007	4.56e-007	4.21e-007
-0.23319	4.31e-007	4.47e-007	3.93e-007
-0.23282	4.56e-007	4.26e-007	3.93e-007
-0.23241	4.29e-007	4.27e-007	4.12e-007
-0.23187	4.12e-007	4.42e-007	4.28e-007
-0.23131	4.2e-007	4.17e-007	3.84e-007
-0.23078	4.22e-007	4.17e-007	4.25e-007
-0.23034	3.96e-007	4e-007	3.8e-007
-0.2298	3.94e-007	4.07e-007	3.74e-007
-0.22944	3.82e-007	4.15e-007	3.82e-007
-0.22874	4.01e-007	3.93e-007	3.85e-007
-0.22824	4.01e-007	3.83e-007	3.66e-007
-0.22784	3.81e-007	3.98e-007	3.49e-007
-0.22743	4.09e-007	3.99e-007	3.82e-007
-0.2269	4.3e-007	3.99e-007	3.74e-007
-0.22635	3.7e-007	3.63e-007	3.48e-007
-0.22598	3.6e-007	3.72e-007	3.48e-007
-0.22535	3.36e-007	3.72e-007	3.75e-007
-0.22489	3.68e-007	3.99e-007	3.57e-007
-0.2243	3.58e-007	3.74e-007	3.42e-007
-0.2239	3.61e-007	3.39e-007	3.35e-007

-0.22329	3.63e-007	3.28e-007	3.3e-007
-0.22295	3.36e-007	3.41e-007	2.79e-007
-0.22241	3.64e-007	3.19e-007	3.22e-007
-0.2218	3.42e-007	3.34e-007	3.16e-007
-0.22134	3.42e-007	3.2e-007	3.24e-007
-0.22075	3.28e-007	3.05e-007	2.95e-007
-0.2204	3.28e-007	3.4e-007	3.01e-007
-0.21992	2.99e-007	2.9e-007	2.89e-007
-0.21933	3.16e-007	3.42e-007	3.23e-007
-0.21888	2.83e-007	2.87e-007	3.14e-007
-0.21844	3.04e-007	3.32e-007	2.99e-007
-0.21797	2.83e-007	3.24e-007	2.91e-007
-0.21747	2.96e-007	2.68e-007	2.63e-007
-0.21688	2.92e-007	3.04e-007	2.72e-007
-0.21633	3.17e-007	2.92e-007	2.77e-007
-0.21588	2.74e-007	3.16e-007	2.75e-007
-0.21556	2.95e-007	3.12e-007	2.65e-007
-0.21496	2.71e-007	3.1e-007	2.42e-007
-0.21445	2.73e-007	2.65e-007	2.58e-007
-0.21386	2.77e-007	2.64e-007	3.03e-007
-0.21338	2.38e-007	2.67e-007	2.8e-007
-0.21296	2.77e-007	2.79e-007	2.74e-007
-0.21244	2.72e-007	2.52e-007	2.63e-007
-0.2119	2.74e-007	3.1e-007	2.88e-007
-0.21153	2.6e-007	2.78e-007	2.75e-007
-0.21097	2.82e-007	2.75e-007	2.91e-007
-0.21039	2.17e-007	2.61e-007	2.77e-007
-0.21009	2.43e-007	2.61e-007	2.6e-007
-0.2095	2.69e-007	2.68e-007	2.26e-007
-0.20897	2.51e-007	2.62e-007	2.54e-007
-0.20844	2.54e-007	2.72e-007	2.39e-007
-0.20804	2.56e-007	2.77e-007	2.77e-007
-0.20762	2.38e-007	2.7e-007	2.63e-007
-0.20699	2.36e-007	2.78e-007	2.77e-007
-0.20652	2.1e-007	2.51e-007	2.54e-007
-0.20606	2.06e-007	2.73e-007	2.48e-007
-0.20548	2.45e-007	2.78e-007	2.57e-007
-0.20496	2.33e-007	2.41e-007	2.47e-007
-0.20444	2.29e-007	2.84e-007	2.59e-007
-0.204	1.91e-007	2.54e-007	2.09e-007
-0.20334	2.33e-007	2.59e-007	2.08e-007
-0.20286	2.12e-007	2.51e-007	2.41e-007
-0.2025	2.05e-007	2.52e-007	1.95e-007

-0.20195	2.34e-007	2.59e-007	2.29e-007
-0.20139	2.15e-007	2.37e-007	2.36e-007
-0.20084	2.35e-007	2.19e-007	2.51e-007
-0.20042	2.35e-007	2.05e-007	2.11e-007
-0.19988	1.93e-007	2.26e-007	2.26e-007
-0.19935	1.89e-007	2.51e-007	2.11e-007
-0.19897	2.03e-007	2.47e-007	2.27e-007
-0.19844	2.18e-007	2.48e-007	2.25e-007
-0.19793	1.88e-007	2.3e-007	2.32e-007
-0.19739	2e-007	2.08e-007	1.78e-007
-0.19693	1.97e-007	2.15e-007	2.16e-007
-0.19661	2.24e-007	2.34e-007	2.3e-007
-0.19593	1.94e-007	2.43e-007	2.28e-007
-0.19542	1.92e-007	2.15e-007	2.35e-007
-0.19478	1.84e-007	1.77e-007	1.94e-007
-0.19438	1.85e-007	2.3e-007	2e-007
-0.19387	1.66e-007	1.81e-007	2.31e-007
-0.1934	2.07e-007	2.42e-007	1.75e-007
-0.19287	1.76e-007	2.08e-007	2.23e-007
-0.19243	2.12e-007	2.31e-007	2.18e-007
-0.192	1.84e-007	2e-007	1.86e-007
-0.19144	1.88e-007	2.33e-007	2.29e-007
-0.19101	1.69e-007	2.29e-007	2e-007
-0.19046	1.94e-007	1.99e-007	2.24e-007
-0.18997	1.95e-007	2.21e-007	1.91e-007
-0.18944	2.03e-007	2.02e-007	2.3e-007
-0.18898	1.61e-007	2.03e-007	2.02e-007
-0.18843	1.95e-007	2.17e-007	2.28e-007
-0.18796	1.67e-007	2.27e-007	2.26e-007
-0.18747	1.58e-007	2.32e-007	2.31e-007
-0.1868	1.58e-007	1.89e-007	2.3e-007
-0.18661	2.23e-007	1.76e-007	1.88e-007
-0.18607	2.07e-007	2.13e-007	2.08e-007
-0.18553	1.67e-007	2.03e-007	2.2e-007
-0.18493	2.03e-007	2.08e-007	1.96e-007
-0.18447	1.64e-007	2.09e-007	1.86e-007
-0.18412	2.02e-007	1.75e-007	2.04e-007
-0.18346	1.73e-007	2.33e-007	1.88e-007
-0.18304	1.69e-007	2.06e-007	2.19e-007
-0.18266	2.2e-007	2.06e-007	2.23e-007
-0.18201	1.72e-007	1.94e-007	2.15e-007
-0.18139	1.55e-007	1.86e-007	1.95e-007
-0.18101	2.02e-007	2.06e-007	2.2e-007

-0.18046	1.71e-007	2.09e-007	2.02e-007
-0.18006	1.54e-007	2.13e-007	1.91e-007
-0.17949	1.8e-007	1.82e-007	2.09e-007
-0.179	1.81e-007	2.27e-007	1.86e-007
-0.17847	1.75e-007	1.63e-007	2.21e-007
-0.17803	1.61e-007	1.73e-007	2.07e-007
-0.17769	1.62e-007	2.14e-007	2.07e-007
-0.17697	2.14e-007	2.22e-007	1.82e-007
-0.17646	1.51e-007	1.85e-007	1.99e-007
-0.17612	1.64e-007	2.21e-007	2.08e-007
-0.17536	1.95e-007	1.9e-007	2.13e-007
-0.17507	2.32e-007	2.14e-007	2.09e-007
-0.17455	2.32e-007	1.85e-007	2.34e-007
-0.17413	1.37e-007	1.95e-007	1.88e-007
-0.1736	1.57e-007	1.93e-007	1.69e-007
-0.17306	1.96e-007	1.91e-007	2.2e-007
-0.17239	1.36e-007	1.95e-007	1.88e-007
-0.17212	1.67e-007	1.88e-007	2.07e-007
-0.17153	1.68e-007	2.04e-007	2.19e-007
-0.171	1.61e-007	1.84e-007	2.14e-007
-0.17054	1.75e-007	2.05e-007	2.02e-007
-0.17017	1.75e-007	2.06e-007	2.14e-007
-0.16954	1.74e-007	1.9e-007	2.07e-007
-0.16898	1.84e-007	2.3e-007	2.07e-007
-0.16861	1.66e-007	1.83e-007	2e-007
-0.16805	1.67e-007	1.85e-007	1.63e-007
-0.16755	1.85e-007	1.84e-007	2.36e-007
-0.16705	1.63e-007	2.11e-007	2.36e-007
-0.16659	1.51e-007	1.95e-007	2.34e-007
-0.16605	1.85e-007	2.13e-007	2.14e-007
-0.16564	1.54e-007	2.02e-007	2.27e-007
-0.16511	1.69e-007	2.28e-007	2.03e-007
-0.16458	1.83e-007	1.96e-007	2.06e-007
-0.16411	1.61e-007	1.67e-007	2.28e-007
-0.16366	1.64e-007	2.21e-007	1.95e-007
-0.16308	1.56e-007	1.75e-007	2.45e-007
-0.16262	1.73e-007	1.76e-007	2.49e-007
-0.16224	1.88e-007	2.01e-007	2.02e-007
-0.16167	1.44e-007	2.22e-007	2.08e-007
-0.16112	1.87e-007	1.96e-007	2.07e-007
-0.16072	1.79e-007	2.05e-007	2.16e-007
-0.16017	1.92e-007	1.78e-007	2.4e-007
-0.15983	1.56e-007	2.24e-007	2.12e-007

-0.15922	1.33e-007	2.11e-007	2.16e-007
-0.15878	1.95e-007	2.16e-007	2.4e-007
-0.15824	1.72e-007	2.01e-007	2.22e-007
-0.15766	1.77e-007	2.09e-007	1.9e-007
-0.15717	1.64e-007	1.92e-007	2.26e-007
-0.15655	1.7e-007	2.24e-007	1.92e-007
-0.15612	1.66e-007	2.32e-007	1.94e-007
-0.1557	1.55e-007	2e-007	1.98e-007
-0.15505	1.68e-007	2.04e-007	2.05e-007
-0.15448	1.4e-007	2.17e-007	2.15e-007
-0.15422	2.03e-007	1.77e-007	2.12e-007
-0.15372	1.53e-007	1.59e-007	2.13e-007
-0.15313	1.66e-007	2.16e-007	2.35e-007
-0.15266	1.7e-007	1.94e-007	2.29e-007
-0.15208	1.76e-007	1.99e-007	2.43e-007
-0.15171	1.41e-007	2.09e-007	1.79e-007
-0.15107	1.68e-007	2.08e-007	1.82e-007
-0.15069	1.76e-007	2e-007	2.19e-007
-0.15023	1.82e-007	2.2e-007	2.4e-007
-0.14966	1.56e-007	1.82e-007	2.23e-007
-0.14916	1.63e-007	1.87e-007	2.04e-007
-0.14866	1.51e-007	2.04e-007	1.95e-007
-0.14837	1.58e-007	2.37e-007	2.29e-007
-0.14758	1.81e-007	2.15e-007	2.2e-007
-0.14718	1.66e-007	2.12e-007	2.11e-007
-0.14646	1.54e-007	1.81e-007	2.11e-007
-0.14625	1.52e-007	2.05e-007	1.74e-007
-0.14561	1.6e-007	2.19e-007	2.34e-007
-0.14521	1.37e-007	2.15e-007	1.93e-007
-0.14471	1.52e-007	2.07e-007	2.21e-007
-0.14416	1.64e-007	2.04e-007	2.11e-007
-0.14376	1.57e-007	1.8e-007	2.13e-007
-0.14328	1.74e-007	2.04e-007	2.49e-007
-0.14274	1.62e-007	1.91e-007	2.19e-007
-0.14227	1.81e-007	1.99e-007	2.12e-007
-0.14169	1.66e-007	1.96e-007	1.7e-007
-0.14114	1.58e-007	1.9e-007	1.9e-007
-0.14081	1.82e-007	2.18e-007	2.39e-007
-0.14026	1.74e-007	1.82e-007	1.87e-007
-0.13964	1.82e-007	2.33e-007	1.86e-007
-0.13915	1.81e-007	2.34e-007	2.38e-007
-0.13865	1.73e-007	1.84e-007	2.04e-007
-0.13821	1.34e-007	2.17e-007	1.97e-007

-0.13763	1.71e-007	2.15e-007	2.13e-007
-0.13722	1.71e-007	2.03e-007	2.09e-007
-0.13661	1.53e-007	1.69e-007	2.21e-007
-0.13622	1.69e-007	2.02e-007	2.12e-007
-0.13582	1.47e-007	2.02e-007	2.03e-007
-0.13513	1.61e-007	2.32e-007	2.2e-007
-0.13466	1.47e-007	2.13e-007	2.01e-007
-0.13427	1.77e-007	1.75e-007	1.86e-007
-0.13384	1.6e-007	2.11e-007	1.98e-007
-0.13328	1.53e-007	2.04e-007	2.05e-007
-0.13272	1.84e-007	1.8e-007	2.04e-007
-0.13218	1.81e-007	2.09e-007	1.81e-007
-0.13162	1.67e-007	1.92e-007	1.81e-007
-0.13113	1.8e-007	2e-007	2.07e-007
-0.13074	1.18e-007	1.86e-007	2.18e-007
-0.13026	1.43e-007	1.99e-007	2.1e-007
-0.12975	1.68e-007	2.08e-007	2.12e-007
-0.12925	1.77e-007	2.19e-007	2.2e-007
-0.12876	1.75e-007	1.97e-007	1.9e-007
-0.12822	1.4e-007	1.78e-007	2.1e-007
-0.12781	1.21e-007	2.15e-007	2.17e-007
-0.12718	1.28e-007	1.81e-007	2.19e-007
-0.12683	1.25e-007	2e-007	2.03e-007
-0.12625	1.81e-007	1.63e-007	1.78e-007
-0.12572	1.59e-007	2.01e-007	1.84e-007
-0.12528	1.65e-007	1.87e-007	1.93e-007
-0.12493	1.7e-007	1.85e-007	1.97e-007
-0.12438	1.42e-007	1.68e-007	2.27e-007
-0.1238	1.75e-007	1.79e-007	1.74e-007
-0.12336	1.56e-007	2.05e-007	2.1e-007
-0.12271	1.33e-007	1.76e-007	1.81e-007
-0.12237	2.07e-007	1.69e-007	2.32e-007
-0.12176	1.55e-007	1.88e-007	2.36e-007
-0.12117	1.41e-007	1.81e-007	1.81e-007
-0.12071	1.52e-007	1.84e-007	1.92e-007
-0.1202	1.68e-007	2e-007	2.25e-007
-0.11972	1.63e-007	1.72e-007	1.82e-007
-0.11923	1.63e-007	1.51e-007	1.95e-007
-0.11873	1.44e-007	1.68e-007	1.94e-007
-0.11821	1.52e-007	2.43e-007	1.72e-007
-0.11769	1.16e-007	1.92e-007	1.5e-007
-0.11727	1.64e-007	1.81e-007	1.56e-007
-0.11661	1.3e-007	2.01e-007	2.25e-007

-0.11624	1.26e-007	1.85e-007	2.05e-007
-0.11571	1.76e-007	1.83e-007	1.98e-007
-0.11528	1.26e-007	1.83e-007	2.23e-007
-0.11464	1.47e-007	1.82e-007	1.74e-007
-0.11416	1.53e-007	1.74e-007	1.7e-007
-0.11371	1.8e-007	1.76e-007	1.68e-007
-0.11324	1.28e-007	2.03e-007	1.68e-007
-0.11272	1.81e-007	2.01e-007	1.97e-007
-0.11221	1.25e-007	1.42e-007	1.75e-007
-0.11166	1.27e-007	2.05e-007	1.6e-007
-0.11127	1.78e-007	1.56e-007	1.99e-007
-0.11076	1.16e-007	1.73e-007	1.83e-007
-0.11021	1.6e-007	1.73e-007	1.95e-007
-0.10961	1.41e-007	1.61e-007	1.76e-007
-0.10904	1.65e-007	1.85e-007	1.92e-007
-0.10861	1.31e-007	1.87e-007	1.29e-007
-0.10821	1.31e-007	1.68e-007	2.04e-007
-0.10768	1.51e-007	1.82e-007	1.48e-007
-0.10727	1.33e-007	1.89e-007	1.67e-007
-0.10669	1.79e-007	1.69e-007	2.01e-007
-0.10612	1.5e-007	1.63e-007	1.8e-007
-0.10563	1.61e-007	1.86e-007	2.17e-007
-0.105	1.38e-007	1.47e-007	1.64e-007
-0.10473	1.29e-007	1.87e-007	1.72e-007
-0.10416	1.42e-007	1.42e-007	1.94e-007
-0.10365	1.5e-007	1.42e-007	1.5e-007
-0.1031	1.25e-007	1.68e-007	1.72e-007
-0.10271	1.34e-007	1.72e-007	1.69e-007
-0.10208	1.38e-007	1.69e-007	1.73e-007
-0.1016	1.55e-007	2.01e-007	1.7e-007
-0.10107	1.62e-007	1.72e-007	1.6e-007
-0.10068	1.43e-007	2.01e-007	2.13e-007
-0.10009	1.78e-007	1.72e-007	1.45e-007
-0.09952	1.59e-007	1.59e-007	1.94e-007
-0.09909	1.82e-007	1.52e-007	1.46e-007
-0.0987	1.25e-007	1.5e-007	1.65e-007
-0.09815	1.32e-007	1.42e-007	1.54e-007
-0.09758	1.36e-007	1.62e-007	1.66e-007
-0.09715	1.33e-007	1.55e-007	1.68e-007
-0.09665	1.18e-007	1.44e-007	1.65e-007
-0.09613	1.33e-007	1.53e-007	1.94e-007
-0.09564	1.14e-007	1.4e-007	1.61e-007
-0.09523	1.26e-007	1.46e-007	1.77e-007

-0.09473	1.43e-007	1.69e-007	1.37e-007
-0.09433	1.12e-007	1.7e-007	1.61e-007
-0.0937	1.38e-007	1.77e-007	1.62e-007
-0.09323	1.33e-007	1.57e-007	1.39e-007
-0.09268	1.34e-007	1.5e-007	1.76e-007
-0.09223	1.39e-007	2.04e-007	1.75e-007
-0.09156	1.36e-007	1.51e-007	1.73e-007
-0.09119	1.49e-007	1.62e-007	1.71e-007
-0.09069	1.15e-007	1.56e-007	1.88e-007
-0.0902	1.21e-007	1.58e-007	1.85e-007
-0.08971	7.58e-008	1.11e-007	1.62e-007
-0.08905	1.35e-007	1.61e-007	1.75e-007
-0.08872	1.64e-007	1.24e-007	1.67e-007
-0.08822	1.18e-007	1.38e-007	1.79e-007
-0.08769	1.45e-007	1.37e-007	1.56e-007
-0.08722	1.23e-007	1.44e-007	1.72e-007
-0.08666	1.12e-007	1.42e-007	2.09e-007
-0.08618	1.37e-007	1.33e-007	1.68e-007
-0.08564	1.61e-007	1.73e-007	1.28e-007
-0.0852	1.45e-007	1.8e-007	1.53e-007
-0.08466	1.63e-007	1.38e-007	1.43e-007
-0.08408	1.47e-007	1.48e-007	1.44e-007
-0.08375	1.72e-007	1.59e-007	1.83e-007
-0.08305	1.54e-007	1.41e-007	1.79e-007
-0.08265	1.09e-007	1.29e-007	1.45e-007
-0.0822	1.37e-007	1.45e-007	1.55e-007
-0.08175	1.06e-007	1.18e-007	1.75e-007
-0.08111	1.37e-007	1.53e-007	1.36e-007
-0.08064	1.36e-007	1.58e-007	1.55e-007
-0.08017	1.33e-007	1.44e-007	1.49e-007
-0.0798	1.39e-007	1.55e-007	1.41e-007
-0.0792	1.42e-007	1.8e-007	1.57e-007
-0.07874	1.08e-007	1.54e-007	1.59e-007
-0.07827	1.52e-007	1.39e-007	1.8e-007
-0.07769	1.45e-007	1.34e-007	1.36e-007
-0.07715	1.38e-007	1.53e-007	1.52e-007
-0.07675	1.35e-007	1.23e-007	1.36e-007
-0.0763	1.37e-007	1.75e-007	1.25e-007
-0.07572	1.37e-007	1.47e-007	1.59e-007
-0.07522	1.19e-007	1.49e-007	1.52e-007
-0.07475	1.49e-007	1.42e-007	1.49e-007
-0.07414	1.21e-007	1.62e-007	1.53e-007
-0.07357	1.43e-007	1.48e-007	1.7e-007

-0.07318	1.38e-007	1.66e-007	1.54e-007
-0.07264	1.42e-007	1.46e-007	1.57e-007
-0.0721	1.23e-007	1.22e-007	1.53e-007
-0.07163	1.65e-007	1.22e-007	1.78e-007
-0.07114	1.51e-007	1.47e-007	1.61e-007
-0.07067	1.51e-007	1.45e-007	1.41e-007
-0.07014	1.32e-007	1.59e-007	1.72e-007
-0.06963	1.21e-007	1.65e-007	1.44e-007
-0.06912	1.27e-007	1.4e-007	1.75e-007
-0.06868	1.33e-007	1.58e-007	1.62e-007
-0.0683	1.26e-007	1.51e-007	1.61e-007
-0.06759	1.17e-007	1.46e-007	2.07e-007
-0.06713	1.48e-007	1.5e-007	1.57e-007
-0.06643	1.44e-007	1.8e-007	1.62e-007
-0.06603	1.45e-007	1.76e-007	1.57e-007
-0.06565	1.48e-007	1.49e-007	1.62e-007
-0.06501	1.24e-007	1.33e-007	1.58e-007
-0.06462	1.33e-007	1.48e-007	1.57e-007
-0.06404	1.3e-007	1.72e-007	1.56e-007
-0.06369	1.56e-007	1.24e-007	1.39e-007
-0.06319	1.18e-007	1.59e-007	1.5e-007
-0.06258	1.11e-007	1.67e-007	1.87e-007
-0.0621	1.42e-007	1.58e-007	1.58e-007
-0.06168	1.45e-007	1.43e-007	1.66e-007
-0.06107	1.36e-007	1.69e-007	1.99e-007
-0.06061	1.31e-007	1.66e-007	1.84e-007
-0.06021	1.17e-007	1.52e-007	1.86e-007
-0.05963	1.41e-007	1.39e-007	1.46e-007
-0.05908	1.17e-007	1.6e-007	1.66e-007
-0.05865	1.69e-007	1.11e-007	1.55e-007
-0.05811	1.13e-007	1.51e-007	1.78e-007
-0.05754	1.41e-007	1.53e-007	1.56e-007
-0.05709	1.7e-007	1.56e-007	1.39e-007
-0.05659	1.26e-007	1.15e-007	1.41e-007
-0.05614	1.39e-007	1.61e-007	1.39e-007
-0.05563	1.42e-007	1.48e-007	1.75e-007
-0.05502	1.41e-007	1.46e-007	1.74e-007
-0.05448	1.38e-007	1.65e-007	1.46e-007
-0.05404	1.24e-007	1.34e-007	1.85e-007
-0.05351	1.28e-007	1.39e-007	1.4e-007
-0.05299	1.52e-007	1.66e-007	1.45e-007
-0.05265	1.51e-007	1.58e-007	1.76e-007
-0.05201	1.47e-007	1.9e-007	1.54e-007

-0.0516	1.36e-007	1.36e-007	1.69e-007
-0.05111	1.46e-007	9.76e-008	1.43e-007
-0.05042	1.2e-007	1.38e-007	1.47e-007
-0.04995	1.42e-007	1.37e-007	1.4e-007
-0.04959	1.51e-007	1.48e-007	1.32e-007
-0.04897	1.15e-007	1.25e-007	1.27e-007
-0.0486	1.41e-007	1.23e-007	1.7e-007
-0.04791	1.53e-007	1.72e-007	1.48e-007
-0.04742	1.49e-007	1.45e-007	1.27e-007
-0.04708	1.27e-007	1.44e-007	1.43e-007
-0.04662	1.41e-007	1.53e-007	1.89e-007
-0.04616	1.37e-007	1.67e-007	1.45e-007
-0.04554	1.51e-007	1.25e-007	1.67e-007
-0.04515	1.24e-007	1.56e-007	1.76e-007
-0.04448	1.5e-007	1.96e-007	1.67e-007
-0.04408	1.38e-007	1.29e-007	1.59e-007
-0.04361	1.4e-007	1.61e-007	1.61e-007
-0.04323	1.17e-007	1.71e-007	1.35e-007
-0.04254	1.29e-007	1.71e-007	1.92e-007
-0.04209	9.54e-008	1.41e-007	1.52e-007
-0.04162	1.6e-007	1.77e-007	1.48e-007
-0.04109	1.6e-007	1.64e-007	1.4e-007
-0.04057	1.6e-007	1.35e-007	1.41e-007
-0.04019	1.17e-007	1.22e-007	1.59e-007
-0.03944	1.55e-007	1.64e-007	1.63e-007
-0.0391	1.29e-007	1.77e-007	1.75e-007
-0.03857	1.36e-007	1.54e-007	1.49e-007
-0.03797	1.53e-007	1.59e-007	1.79e-007
-0.03741	1.15e-007	1.32e-007	1.27e-007
-0.03703	1.33e-007	1.6e-007	1.7e-007
-0.03653	1.46e-007	1.44e-007	1.63e-007
-0.03613	1.41e-007	1.62e-007	1.58e-007
-0.03551	1.43e-007	1.62e-007	1.83e-007
-0.03491	1.56e-007	1.61e-007	1.5e-007
-0.03452	1.39e-007	1.75e-007	1.38e-007
-0.03399	1.4e-007	1.93e-007	1.5e-007
-0.03355	1.4e-007	1.8e-007	1.88e-007
-0.03312	1.3e-007	1.45e-007	2.03e-007
-0.03251	1.31e-007	1.39e-007	1.76e-007
-0.03206	1.61e-007	1.57e-007	1.85e-007
-0.03164	1.46e-007	1.61e-007	1.51e-007
-0.03119	1.45e-007	1.45e-007	1.72e-007
-0.03059	1.4e-007	1.55e-007	1.64e-007

-0.03007	1.13e-007	1.72e-007	1.93e-007
-0.0296	1.67e-007	1.45e-007	1.94e-007
-0.029	1.22e-007	1.83e-007	1.68e-007
-0.02856	1.59e-007	1.4e-007	1.85e-007
-0.02792	1.41e-007	1.77e-007	1.77e-007
-0.02764	1.33e-007	1.47e-007	1.32e-007
-0.02702	1.7e-007	1.7e-007	1.52e-007
-0.02663	1.27e-007	1.59e-007	1.46e-007
-0.02596	1.58e-007	1.58e-007	1.63e-007
-0.02556	1.73e-007	1.44e-007	1.54e-007
-0.02497	1.26e-007	1.89e-007	1.67e-007
-0.02452	1.53e-007	1.73e-007	1.7e-007
-0.02401	1.32e-007	1.51e-007	1.45e-007
-0.02357	1.41e-007	1.91e-007	1.79e-007
-0.02324	1.46e-007	1.67e-007	1.59e-007
-0.02277	1.53e-007	1.29e-007	1.79e-007
-0.02225	1.59e-007	1.81e-007	1.91e-007
-0.02168	1.42e-007	1.69e-007	1.78e-007
-0.02122	1.83e-007	1.69e-007	1.55e-007
-0.02077	1.36e-007	1.42e-007	1.85e-007
-0.02017	1.4e-007	1.71e-007	1.62e-007
-0.01969	1.49e-007	1.26e-007	1.76e-007
-0.01935	1.34e-007	1.61e-007	1.61e-007
-0.01873	1.37e-007	1.68e-007	1.7e-007
-0.01817	1.2e-007	1.82e-007	1.8e-007
-0.01778	1.14e-007	1.63e-007	1.6e-007
-0.01711	1.33e-007	2.01e-007	1.54e-007
-0.01672	1.53e-007	1.48e-007	1.72e-007
-0.0162	1.61e-007	1.95e-007	2.1e-007
-0.01564	1.51e-007	2.09e-007	1.58e-007
-0.01524	1.51e-007	1.75e-007	1.57e-007
-0.01467	1.49e-007	1.59e-007	1.7e-007
-0.0142	1.24e-007	1.74e-007	1.92e-007
-0.01379	1.57e-007	1.59e-007	1.93e-007
-0.01317	1.6e-007	1.69e-007	1.96e-007
-0.01274	9.9e-008	1.98e-007	1.63e-007
-0.01213	1.48e-007	1.5e-007	1.72e-007
-0.01167	1.24e-007	1.63e-007	1.99e-007
-0.01115	1.66e-007	1.59e-007	1.95e-007
-0.01072	1.78e-007	1.93e-007	2e-007
-0.01017	1.19e-007	1.32e-007	1.36e-007
-0.0098	1.7e-007	1.93e-007	1.87e-007
-0.00932	1.43e-007	1.74e-007	1.89e-007

-0.00872	1.3e-007	1.45e-007	1.7e-007
-0.00823	1.82e-007	1.68e-007	1.83e-007
-0.00768	1.39e-007	1.86e-007	1.89e-007
-0.0073	1.63e-007	1.81e-007	1.64e-007
-0.00671	1.23e-007	2.04e-007	1.95e-007
-0.00618	1.36e-007	1.78e-007	2.18e-007
-0.00556	1.43e-007	1.45e-007	2e-007
-0.00513	1.19e-007	1.79e-007	1.85e-007
-0.0047	1.45e-007	1.23e-007	1.98e-007
-0.0043	1.73e-007	2.08e-007	1.89e-007
-0.00355	1.3e-007	1.84e-007	1.39e-007
-0.00312	1.45e-007	2.11e-007	1.84e-007
-0.00278	1.24e-007	1.74e-007	1.61e-007
-0.00227	1.79e-007	1.77e-007	1.58e-007
-0.00163	1.53e-007	1.66e-007	1.71e-007
-0.00114	1.41e-007	1.65e-007	2.14e-007
-0.00062	1.69e-007	1.56e-007	2.16e-007
-0.00017	1.86e-007	1.75e-007	1.67e-007

Table S4. Normalized voltametric peak height titration data for **Figure 3a**.

[ENOX2], nM	Replicate #1	Replicate #2	Trial 3
0.1	9.1e-007	8.9057e-007	9.1236e-007
0.3	9e-007	8.99668e-007	8.99628e-007
1	8.42e-007	8.46173e-007	8.2243e-007
3	7.75e-007	8.09318e-007	7.54015e-007
10	7.23e-007	7.70411e-007	6.80556e-007
30	6.88e-007	7.03925e-007	6.43682e-007
100	6.49e-007	6.70999e-007	6.00069e-007
200	5.98e-007	5.90073e-007	5.59311e-007

Table S5. voltametric data for off-target analysis for **Figure 3b**.

ENOX	BSA	TBP
3.02e-007	7.4e-008	6.6e-008
3.09595e-007	5.3e-008	1.9e-008
3.40317e-007	3.1e-008	1.8e-008
